# Supplementary material for: Predictors of self-reported symptoms and testing for COVID-19 in Canada using a nationally representative survey
Source: PLoS One. 2020 Oct 21;15(10):e0240778. doi: 10.1371/journal.pone.0240778 (PMC7577454; doi:10.1371/journal.pone.0240778)
Supplement: S1 Appendix — (DOCX) [file pone.0240778.s001.docx]

**COVID-19 Questionnaire (Wave 6)**

**(gen pop)**

**[stand-alone screen]**

We have some questions about your life in Canada today.

**QF1.**

**BASE = ALL**

**RANKING (select up to three)**

Please rank the issues facing Canada that you care about the most. Select up to three from the list or write in your own if you do not see the issue you’re thinking of.

**[RANDOMIZE]**

The Deficit / Government spending

The Economy

Health Care

Coronavirus/COVID-19 response

Taxes

Jobs / Unemployment

Environment / Climate Change

Income inequality/Poverty

First Nations/Indigenous issues

Energy / Natural Resources /Pipelines

Ethics / Corruption

Terrorism / Security

Crime / Public safety

National Unity (Western Canada/Quebec)

Immigration/refugees

Housing affordability

International/global issues

Other: (specify)

**QF7.**

**Base=All**

**[single choice grid]**

How concerned are you about each of the following…?

**[rows]**

[T] You personally becoming sick from coronavirus

[T] Someone in your household getting sick

[T] Other family (not in your household) or friends becoming sick

**[columns]**

Not at all concerned

Not that concerned

Moderately concerned

Very concerned

**QF8.**

**Base=All**

**Single choice grid**

Let’s think about the last month or so. In that time have you experienced any of the following symptoms that were NOT related to a condition or illness that you deal with chronically?

[Rows]

1 Difficulty breathing / shortness of breath

2 A fever

3 A mild dry cough

4 A severe dry cough (keeps you from sleeping)

5 Sore throat

6 Frequent sneezing

[Columns]

Yes, had this but it went away

Yes, I still have this

No, have not had this

**QF9.**

**Base = all**

**[single choice]**

To help us with our analysis, please tell us how many people currently live in your household?

Just one – I live alone

Two people

Three people

Four people

Five or more people in my household

**QF10.**

**Base: Exclude living alone in QF4**

**[Single choice grid]**

Thinking about the last month or so in your household, has anyone else experienced any of the following symptoms that were NOT related to a condition or illness they deal with chronically?

[Rows]

1 Difficulty breathing / shortness of breath

2 A fever

3 A mild dry cough

4 A severe dry cough (keeps you from sleeping)

5 Sore throat

6 Frequent sneezing

[Columns]

Yes, someone had this, but it went away

Yes, someone still has this

No, no one has had this

**QF11A.**

**Base: All**

**[Single choice**]

When it comes to COVID-19, which of these scenarios best describes your experience:

1 I have been tested for COVID-19

2 I am scheduled to be tested

3 Trying to get tested but haven’t been able to

4 Have done a self-assessment through government website/app

5 Not tested nor planning to be tested or self assessed

**QF11B.**

**Base those who have tested = 1**

**[Single choice]**

Thinking about the process of getting tested, would you say the time that it took to get tested was…?

Reasonable

Unreasonable

**QF12.**

**Base those who are waiting/trying to access a test = 2+3**

**[Single choice]**

You mentioned that you are awaiting testing or trying to get it. Thinking about this process and the circumstances, would you say the amount of time it is taking to get tested is…?

Reasonable

Unreasonable

**QF13.**

**Base – have been tested = 1**

**[Single choice]**

And what about the time that it took or is taking for you to receive your test results – is this amount of time…?

Reasonable

Unreasonable

**QF14.**

**Base: Exclude living alone in QF9**

**[Single choice**]

What about others in your household, has anyone else been tested?

1 Someone else has been tested for COVID-19

2 They are scheduled to be tested

3 They are trying to get tested but haven’t been able to

4 They’ve done a self-assessment through government website/app

5 Not tested or planning to be/Not tested, they feel fine

**QF15.**

**Base those who have had someone else in their home tested = 1**

**[Single choice]**

Thinking about the process of getting tested, would you say the time that it took for this other person in your home to get tested was…?

Reasonable

Unreasonable

**QF16.**

**Base those who have someone else in their home waiting/trying to access a test = 2+3**

**[Single choice]**

You mentioned that another person in your home is awaiting testing or trying to get it. Thinking about this process and the circumstances, would you say the amount of time it is taking for them to get tested is…?

Reasonable

Unreasonable

**QF17.**

**Base – those who have had someone else in their home been tested = 1**

**[Single choice]**

And what about the time that it took for this person to receive their test results – was this amount of time…?

Reasonable

Unreasonable

**A1.**

**Base=ALL**

**[MULTI-CHOICE, SELECT ALL THAT APPLY]**

Many of us have more free time these days. Is there anything you’ve been doing more in the last few weeks? Please choose all that apply:

Playing board games/doing puzzles

Reading books

Playing video games

Baking/cooking

Streaming shows and movies (Netflix, Crave, Apple TV etc.)

Going for walks

Exercising in other ways than walks

Eating unhealthy food

Phone/Video calling friends and family

Cleaning/organizing your space

Other: specify___________

**A2.**

**Base=All**

**[Single choice]**

Based on whatever you’ve read, seen or heard, when do you anticipate things will be “back to normal” in Canada?

A few weeks

A month or two

Three to six months

Six months to a year

Later than the end of 2021

**Q24.**

**Base = All**

**[MULTI-CHOICE, MAX 2]**

When things get “back to normal”, whenever that is, what are you MOST looking forward to doing. Please select up to two:

Hugging friends/family outside your household

Reconnecting with people I haven’t seen

Going on vacation

Going to a restaurant/café/bar

Going to the gym/a fitness class

Going to a public event i.e. hockey game/concert etc.

Going back to work/workplace

Watching live sports

Going shopping

Other: (specify)
