# Supplementary material for: Predictors of self-reported symptoms and testing for COVID-19 in Canada using a nationally representative survey
Source: PLoS One. 2020 Oct 21;15(10):e0240778. doi: 10.1371/journal.pone.0240778 (PMC7577454; doi:10.1371/journal.pone.0240778)
Supplement: S2 Appendix — (DOCX) [file pone.0240778.s002.docx]

# **ABSTRACT: The Ab-C Study**

There is an urgent need to understand how many Canadians have been infected with SARS-CoV-2, the virus that causes coronavirus disease (COVID-19). The most rigorous scientific way to establish infection rates is to conduct a random blood-based survey of Canadians to capture a representative profile of the population. The Action to beat Coronavirus (Ab-C) Study proposes to work with reputable polling companies to survey a random sample of about 10,000 Canadians over the age of 18, with an oversample of adults aged 60 or higher to ask about self-reported COVID-19 symptoms and health seeking behaviour, and to seek permission for re-contact to obtain a small amount of blood (using a safe, well-tested home-based blood spot collection systems). The blood would be then sent safely to central laboratories for short-term storage, and then tested with the state-of-the-art assays to detect antibodies to SARS-CoV-2, which indicates a past infection (which may or may not have been symptomatic).

Each participant who provides blood will be given their result in a confidential, secure way with results stratified as “very likely infected in the past,” or “somewhat likely to have past infection,” or “unlikely to have past infection.” This reporting is consistent with the current uncertainties in the test results.

A small subsample (500-700) of those who complete the questionnaire and return a blood spot sample will be re-contacted within weeks of receiving their sample, to investigate a separate question about household-level viral spread. This will involve recruiting another adult in the household to participate, using the same methods, with the aim of investigating antibodies in pairs of both symptom concordant (both household members either had or did not have COVID-19 symptoms) and symptom discordant (one had symptoms, one did not) household members. The subsample will be drawn randomly based on symptom responses on index participants’ questionnaires.

An additional convenience sample of up to 500 participants will be drawn from the several hundred direct requests received by the study team to participate in the study. Those with unusual exposures or other characteristics (e.g., passengers from the Diamond Princess cruise ship) will be invited to participate in the questionnaire (the same questionnaire but sent directly from St. Michael’s rather than Angus Reid) and blood spot collection.

All participants would be asked to provide another sample in July/August, to establish trends in infection (including those who were infected losing antibodies, and those uninfected initially becoming positive. This would provide a unique look into the population-based immunity trends.

The full testing strategies, including location of testing will be developed in close collaboration with Public Health Ontario, the National Microbiological Labs and other key experts. Contact will also be made with other major antibody studies in Canada and around the world, a goal being to harmonize methods to the extent possible, so that results can be compared reliably.

The age- and sex-specific prevalence of antibodies will provide crucial information to understand how many Canadians have been infected, which age and key demographic groups are the most affected, and to establish trends in infection. This information paired with existing data on hospitalization and death rates can be used to guide public health responses against COVID-19.

# **RATIONALE AND HYPOTHESIS/RESEARCH QUESTION**

## **What is the rationale for this study?**

About three months after the first cases of COVID-19 were detected in Canada, there remains considerable uncertainty about the number of people who have been infected with COVID-19. Diagnostic testing for the virus using PCR methods has increased substantially, but a recent poll found only 2% of Canadians had been tested, including only 10% of those with possible COVID-19 symptoms (<https://www.scribd.com/document/456441693/COVID19NewsRelease-14April2020-FINAL>).

Moreover testing has well established biases in who gets tested. The most reliable method to document the prevalence of past infection in the population is to conduct a random survey of Canadians and to test their blood to document past infection.

## **Study hypotheses**

How many Canadians have been infected with SARS-CoV-2? How does this prevalence vary by age and sex? Will this prevalence change over time? What is the relationship between self-reported COVID-19 symptoms among household members and antibody prevalence?

## **Significance**

We will be able to estimate the number of people with detectable antibodies to SARS-COV2 from a random sampling of adults. We will also be able to monitor the trends in the prevalence of infection over time, shed light on household transmission dynamics, and contribute to the understanding of the relationship between antibodies and immunity.

Reliable measurement of past infection will be crucial to establishing the age- and sex-specific infection rates and to estimate the infection fatality rate (IFR). Both types of data can vastly improve control strategies, and provide information to inform the public about the status of the epidemic.

We will oversample older adults (age 60 or higher) to better understand the prevalence rates in that most-vulnerable group and the relationship of infection with the apparently steep rise in hospitalization and death rates at older ages.

# **DESIGN AND METHODOLOGY**

Ab-C is a large, simple survey of Canadians over the age of 18 years. It has a two-part survey method. The first will be a very brief opinion poll of households already enrolled in the Angus Reid Forum Panel (<http://www.angusreidforum.com/>). This is a nationally representative panel of 50,000 Canadians used for political and other social polling. It has recently also surveyed 4,240 Canadians to determine the prevalence of self-reported COVID-19 symptoms (<http://angusreid.org/covid-epidemiology-study/>). That study found about 8% of Canadians reported a combination COVID-19 symptoms.

**Phase 1:** The Angus Reid Institute will implement the brief opinion poll among about 15,000 adults in Canada over the age of 18 years, based on their existing demographic information. Respondents will be asked about COVID-19 symptoms. At the end of the poll, respondents will be asked their permission for the Ab-C team from St. Michael’s Hospital to re-contact them, and to share their polled results with the Ab-C team.

**Phase 2** will involve mailing a blood collection kit to all those who agree after phase 1. The kit will include a disposable lancet (spring-loaded for safety), special filter paper (barcoded) or microcapillary tube, and return materials (Ziplock bag for the sample containing silica gel tablet; and a large plastic bio-safe return pre-paid envelope). During this phase, participants will be asked if they would like the results of the blood testing (Yes, No) explaining that the serology results are not suitable for any clinical decisions.

A subsample of 500-700 participants living with at least one other adult will be drawn from the participants providing dried blood samples, representing four types of pairs: 1) both positive for COVID-19 symptoms, 2) both negative for COVID-19 symptoms, 3) index respondent positive, other adult negative for COVID-19 symptoms, and 4) index respondent negative, other adult positive for COVID-19 symptoms. Emails will be sent to the index respondents asking for permission to contact the other household member, and asking them to provide contact information. The other adult will be given the choice of contacting the research team directly or being contacted by the team. The same process as for index participants will be followed for those consenting, including the questionnaire and dried blood sample collection.

**Phase 3** will involve central testing of all collected dried blood spot samples (DBS). The choice of the exact ELISA will be driven by forthcoming reviews by the Public Health Agency of Canada’s National Microbiological Laboratory (NML) and Public Health Ontario (PHO). Very likely, the assay(s) will measure IgG and IgM, suggesting both distant and more recent infection. We will include IgA as a measure of mucosal immunity/exposure where possible. We will also consult with colleagues conducting ELISA tests as part of a US NIH Study (<https://clinicaltrials.gov/ct2/show/NCT04334954>), and a planned population-based survey in Ohio (Sam Clark, personal communication), a recent Californian survey (<https://www.medrxiv.org/content/10.1101/2020.04.14.20062463v1>), and the results of ongoing testing in Europe using antibody assays (www.serotracker.com). The final assay (s) will be assessed for their sensitivity and specificity including with any cross-comparative studies across the above sites. <https://www.nature.com/articles/d41586-020-01095-0>

**Phase 4** will return the results of the assay to each participant who asked for their result in a confidential, secure way. Results will be stratified as “very likely infected in the past,” “somewhat likely to have past infection,” or “unlikely to have past infection.” This reporting is consistent with the current uncertainties in the test results. We will also ask permission for repeat contact at this time

**Phase 5** will return to the cohort in 4-5 months and ask if they would consent again to re-interview and another home DBS sample. This then would help establish trends in population immunity/exposure.

## **Primary outcome measures/goals of the study**

The number of Canadian adults with detectable antibodies to SARS-COV2 from a random sampling of adults (Phase 1-4). We will also be able to quantify the change in antibodies over time (Phase 5), including persistence of any immunity. The subsample will provide information on the relationship between symptoms, household viral spread, and antibodies in household members.

## **Eligibility**

INCLUSION CRITERIA:

- 1. >= 18 years of age.
  2. Willing and able to complete a verbal telephone consent.
  3. Willing to undergo one blood draw or home blood sampling (asked during Phase 1).
  4. Willing to have blood samples stored for future research.

EXCLUSION CRITERIA:

1. Refused permission in Phase 1 for home blood sampling.
2. Inability to complete questionnaires in English or French.

## **Procedures**

See Appendix A for the initial telephone polling questionnaire

## **Blood collection kits**

For each adult who agrees to participate, we will generate a confidential bar code and secure 8-digit “strong” user ID. These will be attached to the filter paper (a special version of Whatman high quality filter paper that is stable for storage at -20 Celsius for prolonged periods) as well as the written consent form.

The package will be sent by mail to each person at their home address. See Appendix C for a set of instructions and a video at <https://abcstudy.ca/participants/>

The package will comprise:

1. Instructions in English/French in plain language on how to do home blood testing. This will include links to web materials on how to collect the sample.
2. Alcohol swab and gauze.
3. Spring loaded lancet “pen” to enable puncture of fingertip, which retracts after use (for safe disposal).
4. Filter paper (or if we decide, a capillary tube that “auto-draws” the sample) with four circles each of about 0.5cm diameter for 4 blood spots.
5. Ziplock bag with silica gel to counter moisture.
6. Information sheet about the study (English).
7. Consent form.
8. Plastic, tamper-proof, and bio-safety sealed pre-paid mailing envelope in which to return the DBS, consent form (unless internet version is used) and the used lancet.

**Safety:** Like a blood draw at a doctor’s office, risks of at-home blood sampling include discomfort, bruising and, rarely, fainting or infection at the site where the lancet enters the skin. The lancet is designed to prevent full-length puncture of the fingertip for example. The kit includes bandages and gauze to reduce the risk of infection. There is no evidence that SARS-CoV-2 can be transmitted by blood, in the case that one person is helping another in the household. As well, it also is safe to ship these kits in the mail. There is no risk of bloodborne SARS-CoV-2 transmission to the mail carrier or others who handle the package. Similar biological samples are used by the Ontario Government for stool testing, and Blood Canada uses similar mailed samples for bone marrow donation programs.

## **Sample sizes for varying sensitivity and specificity**

Based on preliminary data from the Netherlands and from Santa Clara, California (<https://www.nature.com/articles/d41586-020-01095-0>), we estimate that the true prevalence of infection in Canada is about 3% among all adults, and as-yet uncertain percentage at age 60 and above (Data from Alberta among tested population suggest that prevalence is about 30% lower than overall at ages 60-79 but rises sharply above age 80 years- <https://covid19stats.alberta.ca/>). Hence, we are aiming to sample about 6000 adults age 20-59 years in an age distribution representative of Canada’s population, and about 4000 adults aged 60 and above.

For the 6000 adults below age 60, assuming a precision of 2%, and with 99% confidence intervals, the range of sensitivity (se) and specificity (sp) values are shown below. This suggests that provided sensitivity does not fall below 80% and (more importantly) specificity does not fall below 95%, the survey should be able to detect the true prevalence of infection in adults at these ages reliably (shown in italics). Given the uncertainties about prevalences at older ages, the 4000 samples planned would be sufficient with similar sensitivities and specificity thresholds (shown in italics). The experience to date from various antibody studies ([www.serotracker.com](http://www.serotracker.com)) finds that sensitivity and specificity will be in the range of 90-95% and 98-99%, respectively. This means that we should expect the number of true positives to far exceed the number of false positives. Even with misclassification of assays, the comparison of relative risks in key strata (e.g., with/without symptoms, or with/without household exposure, and subsets of IgA/IgG/IgM) should remain unbiased.

| \|  \| **Se = 0.8** \| **Se = 0.9** \| **Se = 0.95** \| **Se = 0.99** \| **Se = 0.999** \| \| --- \| --- \| --- \| --- \| --- \| --- \| \| Sp = 0.95 \| *1983* \| *1603* \| *1456* \| *1354* \| *1332* \| \| Sp = 0.99 \| *866* \| *741* \| *690* \| *654* \| *647* \| \| Sp = 0.999 \| *633* \| *558* \| *527* \| *505* \| *500* \| |
| --- | --- | --- | --- | --- | --- | --- | --- | --- | --- | --- | --- | --- | --- | --- | --- | --- | --- | --- | --- | --- | --- | --- | --- | --- |

Table 1 : Sensitivity/specificity scenarios and required sample size.
Source: <https://epitools.ausvet.com.au/prevalencess>

# **ANALYSIS**

The analyses of the Phase 1 data will establish age and sex specific prevalences of self-reported COVID-19 symptoms, stratified by neighbourhood income (based on postal code), and testing access (including testing by other household members).

The main analyses will be simple age- and sex-specific prevalences of SARS-CoV-2 taking into account the sensitivity and specificity of the assay(s) as well as the sampling probability of the Angus Reid Forum (called “sample weights”).

The results, with enough positives, can be stratified by sex, age, broad SES (postal code), past COVID-19 symptoms or not, and also health seeking behaviour (e.g. someone else in the household who was tested). We will examine results separately for those with/without COVID-19 symptoms or a family member with these symptoms (or who got tested), those in higher risk professions or who have recently visited a long-term care facility or hospital. We will examine prevalences separately above and below age 60. If sufficient people test positive, the analyses will also examine differences across geographic areas. We will also examine the demographic details of those declining the DBS and compare these distributions to overall Census Canada information to establish representativeness by neighbourhood income and other SES groups.

The technical details of the DBS laboratory analyses awaits further development of the NML/PHO testing strategies and global development of increasingly reliable antibody tests. The properties of the test or tests ultimately chosen, which are not yet known, will guide the interpretation of results.

Pair-wise analyses of the symptom concordant and discordant household members will compare antibody results with self-reported symptomatology.

Further analyses will be of multivariate predictors of positivity and other statistical analyses as appropriate using standard logistic regression models.

# **Ab-C Study Investigators**

St. Michael’s Hospital (Unity Health Toronto) (Ab-C Secretariat):

Prabhat Jha, Director CGHR and Scientist (PI)

Heyu Ni, Scientist

Arthur Slutsky, Scientist

Gillian Booth, Scientist

Patrick Brown, Scientist

Peter Juni, Scientist, Director AHRC

Hellen Gelband, CGHR Senior Fellow

Nico Nagelkerke, CGHR Senior Fellow

Eric Young, CGHR Training coordinator

Abha Sharma, CGHR PDF

Peter Rodriguez, CGHR Database Coordinator

Rajeev Kamadod, CGHR IT Consultant

Maria Pasic, Lab Scientist
Ron Weingust, Lab Scientist

Chaim Birnboin, Senior Technical Advisor

University Health Network

Isaac Bogoch, Scientist

Karla Fisher, PDF

Public Health Ontario

Vanessa Tran

Children’s Hospital of Eastern Ottawa

Pranesh Chakraborty

External partners (not listed on the IRB)

Angus Reid

Ed Morawski

Demetre Eliopoulos

Teresa Lam

# **Appendix A: POLLING QUESTIONAIRRE**

**Part 1 – Introduction + Screener
Part 2 – General
Part 3 – Symptoms
Part 4 – Testing History
Part 5 - Profiling
Part 6 – In-home Test invitation & Conclusion**

## **PART 1 [MAIN ANGUS REID COHORT] – Introduction & Screener**

**[VERSION 1]**

Dear Angus Reid Forum member,

Thank you for your participation in today’s survey!

Let’s begin!

## **PART 1 [HOUSEHOLD SUBSTUDY COHORT] – Introduction & Screener**

**[VERSION 1]**

Dear Participant,

Thank you for joining the Action to Beat Coronavirus (Ab-C) Study today! As you know, you were asked to participate because another member of your household has already completed this survey and provided a dried blood spot sample. We have asked you and others to enrich the ability of the study to learn about household viral transmission.

Let’s begin!

## **PART 1 [CONVENIENCE SAMPLE] – Introduction & Screener**

**[VERSION 1]**

Dear Participant,

Thank you for your interest in volunteering to join the Action to Beat Coronavirus (Ab-C) Study through your email or phone call to us. Your results will help us better understand who has had COVID-19 in Canada, and how it was transmitted.

Let’s begin!

## **PART 2 - General**

**QF7.**

**Base=All**

**[single choice grid]**

How concerned are you about each of the following related to the coronavirus…?

**[rows]**

You personally becoming sick from coronavirus

Someone in your household getting sick

Other family (not in your household) or friends becoming sick

**[columns]**

Not at all concerned

Not that concerned

Moderately concerned

Very concerned

## **PART 3 – Information and Symptoms**

What year were you born?

**QF8.**

**Base=All**

**Single choice grid**

Since the beginning of March, have you experienced any of the following symptoms that were NOT related to a condition or illness that you deal with chronically?

**[Rows]**

Difficulty breathing / shortness of breath

A fever

A mild dry cough

A severe dry cough (keeps you from sleeping)

Sore throat

Frequent sneezing

Loss of sense of smell or taste

Fever with hallucinations

*On >age 60 only*

Unusual or disturbed sleep

Loss of appetite

Dizziness

**[Columns]**

Yes, had this but it went away

*If YES, was this in March or April*

Yes, I still have this

No, have not had this

**QF9.**

**Base = all**

**[single choice]**

To help us with our analysis, please tell us how many people currently live in your household including you?

Just one – I live alone

Two people

Three people

Four people

Five or more people in my household

**QF9a.**

**Base = IF TWO OR MORE IN QF9**

**[single choice]**

How many of the people currently living in your household are 60 years of age or older?

One

Two

Three

Four

Five or more

**QF10.**

**Base: Exclude living alone in QF4**

**Single choice grid**

Since the beginning of March, has anyone else in your household experienced any of the following symptoms that were NOT related to a condition or illness they deal with chronically?

[Rows]

1 Difficulty breathing / shortness of breath

2 A fever

3 A mild dry cough

4 A severe dry cough (keeps you from sleeping)

5 Sore throat

6 Frequent sneezing

7 Loss of sense of smell or taste

8 Fever with hallucinations

*On >age 60 only*

Unusual or disturbed sleep

Loss of appetite

Dizziness

[Columns]

Yes, someone had this, but it went away

If Yes, was this in March or April

Yes, someone still has this

No, no one has had this

## **PART 4 – Testing History**

**QF11A.**

**Base: All**

**Single choice**

When it comes to COVID-19, which of these scenarios best describes your experience:

I have been tested for COVID-19

I am scheduled to be tested

Trying to get tested but haven’t been able to

Have done a self-assessment through government website/app

No, I have not been tested

**QF14.**

**Base: Exclude living alone in QF9**

**Single choice**

What about others in your household, has anyone else been tested?

Someone else has been tested for COVID-19

They are scheduled to be tested

They are trying to get tested but haven’t been able to

They’ve done a self-assessment through government website/app

Not tested or planning to be/Not tested, they feel fine

## **PART 5 – Profiling**

**G1.**

**Base=ALL**

**SINGLE CHOICE**

Now we have a few questions about you.

**G2.**

**Base=ALL**

**SINGLE CHOICE**

What is your sex (or the sex that you were assigned at birth)

Male

Female

Prefer not to say

**G2.**

**Base=ALL**

**SINGLE CHOICE**

How would you rate your own general health?

Excellent
Good
Average
Poor
Don’t know

Prefer not to answer

**G2.**

**Base=ALL**

**SINGLE CHOICE**

How tall are you? Please indicate whether you’d like to answer in Feet or cm.

Feet

Cm

Prefer not to answer

**G2_FEET**

**Base=FEET in G2**

**SINGLE CHOICE**

How tall are you?

**INSERT FEET SCALE**

**G2_CM**

**Base=CM in G2**

**SINGLE CHOICE**

How tall are you?

**INSERT CM SCALE**

**G3**

**Base=ALL**

**SINGLE CHOICE**

How much do you weigh? Please indicate whether you’d like to answer in pounds or kilograms

Pounds

Kilograms

Prefer not to answer

**G2_POUNDS**

**Base=POUNDS in G2**

**SINGLE CHOICE**

How much do you weigh?

**INSERT FEET SCALE**

**G3_KILOGRAMS**

**Base=KILOGRAMS in G3**

**SINGLE CHOICE**

How much do you weigh?

**INSERT KILOGRAM SCALE**

**G4**

**Base=TOTAL**

**SINGLE CHOICE**

How would you describe your current smoking status?

I never smoked

I smoke daily or occasionally

I used to smoke but quit

Don’t know

Prefer not to answer

**G5**

**Base=ALL**

**SINGLE CHOICE**

Has a doctor ever diagnosed you as a diabetic?

Yes

No

Don’t know

Prefer not to answer

**G6**

**Base=TOTAL**

**SINGLE CHOICE**

Has a doctor ever diagnosed you as having high blood pressure?

Yes

No

Don’t know

Prefer not to answer

**For yes only:**

If you are on any drugs for your blood pressure, could you write the name of it here

____________________text

No drugs

Do not know

Prefer not to answer

**G7**

**Base=ALL**

**SINGLE CHOICE GRID**

**Since February 1st, 2020,** have you worked in any of these professions?

**ROWS**

Any health care (Nurse, Doctor, Dentist, work in a health care or long-term nursing/care facility)

Grocery clerk or pharmacy employee

Firefighter or police officer

Flight attendant

Transit driver (including tax and ride-sharing like Uber/Lyft)

Restaurant employee

Gas station employee

**COLUMNS**

Yes

No

Prefer not to answer

**G8**

**Base=ALL**

**SINGLE CHOICE GRID**

**Since February 1st, 2020,** have you visited any of the following facilities:

**ROWS**

Doctor’s office

Emergency Room

Nursing home

**COLUMNS**

Yes

No

Prefer not to answer

## **PART SIX – In-Home Testing Invitation & Conclusion**

SCREEN 1 [FOR MAIN ANGUS REID COHORT]

Dear Angus Reid Forum member,

We have a special request during this time when our country continues to manage the Covid-19 pandemic.

This survey is the foundation of a unique research partnership between **Angus Reid** and **St. Michael’s Hospital** in Toronto.  This is called the “**Action to Beat Coronavirus in Canada**” or the **Ab-C study**.

One of the core questions that has eluded health and government officials around the world is: *what is the true incidence of Covid-19 infection?* In other words – how many people have been exposed to the virus?

The answer to this question will help guide our course of action in this country. This research initiative is aimed at helping us gain this knowledge.

We are asking only a subset of randomly selected members of the Angus Reid Forum like yourself, regardless if you have had symptoms or not, if you would be willing to take part in an at home blood test that will be mailed to you by St. Michael’s Hospital. Every measure has been taken to ensure this process is simple and safe.

St Michael’s Hospital will share the results of the test with you (Confidentially of course). The results will tell you if it is very likely, somewhat likely or unlikely that you have been infected with the virus in the past.

We also have a special appeal from **XXXXX** at the following link **[ENTER APPEAL LINK].**

The video link below will show you exactly what you have to do to complete the test. [**INSERT BLOOD TEST VIDEO LINK]**

I thank you in advance for your participation.

Warm regards and stay safe

**[SIGNATURE]**

Angus Reid

**[PICTURE]**

**YES**, I would like to learn more about this test **[SKIP TO CONSENT PAGE]**
**NO**, I am not interested **[SKIP TO SURVEY END]**

SCREEN 1 [FOR HOUSEHOLD CONTACT SUBSTUDY]

Dear Fellow Canadian,

We have a special request during this time when our country continues to manage the Covid-19 pandemic.

This survey is the foundation of a unique research partnership between **Angus Reid** and **St. Michael’s Hospital** in Toronto.  This is called the “**Action to Beat Coronavirus in Canada**” or the **Ab-C study**.

One of the core questions that has eluded health and government officials around the world is: *what is the true incidence of Covid-19 infection?* In other words – how many people have been exposed to the virus?

The answer to this question will help guide our course of action in this country. This research initiative is aimed at helping us gain this knowledge.

A member of your household was recruited by the Angus Reid Forum, of which they are a member, and has already completed the questionnaire that you just completed. They have also provided the study team at St. Michael’s Hospital with a dried blood spot sample, to be analyzed for coronavirus antibodies. This sub-study, of which you are a part, will help us understand the household dynamics of coronavirus spread. Regardless of whether you have had symptoms or not, would you be willing to do the same at-home blood test as the other person in your household? If you agree, the team at St. Michael’s Hospital will mail you the blood collection kit. Every measure has been taken to ensure this process is simple and safe.

St Michael’s Hospital will be pleased to share the results of your test with you (confidentially, of course). The results will tell you if it is very likely, somewhat likely or unlikely that you have been infected with the virus in the past.

The video link below will show you exactly what you have to do to complete the test. [<https://abcstudy.ca/participants/>**]**

I thank you in advance for your participation.

Warm regards and stay safe

**[SIGNATURE]**

**YES**, I would like to learn more about this test **[SKIP TO CONSENT PAGE]**
**NO**, I am not interested **[SKIP TO SURVEY END]**

SCREEN 2

Thank you for your continued interest in this vital public health initiative. The following link provides you with more information about the test, its sponsors (St. Michaels Hospital in Toronto) as well as a 1-800 information line you are free to call to speak with a representative of the St. Michaels Hospital research team.

**LINK to LANDING PAGE (**[**www.abcstudy.ca**](http://www.abcstudy.ca)**)**

**CONSENT1**

**Base=ALL**

**SINGLE CHOICE**

Do you agree to share your above answers from the first part with the Ab-C research team, who will send you a kit with simple instructions to let you do a self-administered home blood test?

Yes, I agree to participate. Please send me the self-administered home blood test

No, I do not want to participate in the blood test.

**IF NO SKIP TO SURVEY END
IF YES LINK TO ADDRESS INTAKE FORM THEN TO SCREEN 3**

SCREEN 3

Can you please provide us with the following information:

**Name Last, First**

**Address**

**City, Province**

**Postal Code**

**Email**

**Phone**

Thank you. A kit in your name will be sent soon. You should receive it within the next 14 days. For any questions you can contact us at 1 833 TEST ABC (837 8222) or see the website www.abcstudy.ca

**CONCLUSION WITH THANKS:**

**STANDARD SURVEY END PAGE**
