# Supplementary material for: Predictors of self-reported symptoms and testing for COVID-19 in Canada using a nationally representative survey
Source: PLoS One. 2020 Oct 21;15(10):e0240778. doi: 10.1371/journal.pone.0240778 (PMC7577454; doi:10.1371/journal.pone.0240778)
Supplement: Attachment — Submitted filename: Response to reviewers.docx [file pone.0240778.s003.docx]

**Revision to submission [PONE-D-20-16713]: Determinants of self-reported symptoms and testing for COVID-19 in Canada using a nationally representative survey**

**Comment 1-** Please ensure that your manuscript meets PLOS ONE's style requirements, including those for file naming. The PLOS ONE style templates can be found at <https://journals.plos.org/plosone/s/file?id=wjVg/PLOSOne_formatting_sample_main_body.pdf> and <https://journals.plos.org/plosone/s/file?id=ba62/PLOSOne_formatting_sample_title_authors_affiliations.pdf>

**Response 1- We have edited the manuscript to meet PLOS ONE’s style requirements.**

**Comment 2-** We note that you have included the phrase “data not shown” in your manuscript. Unfortunately, this does not meet our data sharing requirements. PLOS does not permit references to inaccessible data. We require that authors provide all relevant data within the paper, Supporting Information files, or in an acceptable, public repository. Please add a citation to support this phrase or upload the data that corresponds with these findings to a stable repository (such as Figshare or Dryad) and provide and URLs, DOIs, or accession numbers that may be used to access these data. Or, if the data are not a core part of the research being presented in your study, we ask that you remove the phrase that refers to these data.

**Response 2- We have removed those statements with the phrase “data not shown”, as these results were not a core part of the research being presented in our study.**

**Comment 3-** Please clarify your Data availability statement. Please note that PLOS ONE criteria require that authors make all data underlying the findings described in their manuscript fully available without restriction at the time of publication. Authors should share any data specific to their analysis that they can legally distribute. For data that they cannot legally distribute, the authors must include in the Data Availability Statement all necessary contact information where an interested researcher would need to apply to gain access to the data; we do not allow an author to be the only point of contact for fielding requests for access to restricted data (for more information, please see https://journals.plos.org/plosone/s/data-availability).

We note that you have indicated that data from this study are available upon request. PLOS only allows data to be available upon request if there are legal or ethical restrictions on sharing data publicly. For information on unacceptable data access restrictions, please see <http://journals.plos.org/plosone/s/data-availability#loc-unacceptable-data-access-restrictions>.

**Response 3- We have changed the Data availability statement (line 121-126).**

In your revised cover letter, please address the following prompts:

a) If there are ethical or legal restrictions on sharing a de-identified data set, please explain them in detail (e.g., data contain potentially identifying or sensitive patient information) and who has imposed them (e.g., an ethics committee). Please also provide contact information for a data access committee, ethics committee, or other institutional body to which data requests may be sent.

b) If there are no restrictions, please upload the minimal anonymized data set necessary to replicate your study findings as either Supporting Information files or to a stable, public repository and provide us with the relevant URLs, DOIs, or accession numbers. Please see http://www.bmj.com/content/340/bmj.c181.long for guidelines on how to de-identify and prepare clinical data for publication. For a list of acceptable repositories, please see http://journals.plos.org/plosone/s/data-availability#loc-recommended-repositories.

We will update your Data Availability statement on your behalf to reflect the information you provide.

**Response- We have revised the cover letter to reflect the legal and ownership restrictions on sharing de-identified dataset.**

**Comment 4-**  Please confirm whether a waiver of Ethics approval was obtained."

**Response 4- IRB approval for this study was obtained from Unity Health Toronto Research Ethics Board (REB# 20-107).**

**Comment 5-** In your Methods section, please provide additional information about the participant recruitment method and the demographic details of your participants. Please ensure you have provided sufficient details to replicate the analyses such as: a) the recruitment date range (month and year),

**Response 5- We have provided the recruitment date range- April 1-5, 2020. Demographic details of the participants are given in the Results section in Table 1.**

b) a description of any inclusion/exclusion criteria that were applied to participant recruitment,

**Response- We have now provided the inclusion/exclusion criteria for participant recruitment (lines 62-63)**

c) a table of relevant demographic details,

**Response- We have included a table of relevant demographic details as Table 1.**

d) a statement as to whether your sample can be considered representative of a larger population,

**Response- We have included the statement: “The Angus Reid Institute (ARI) conducted an online survey from April 1-5, 2020, among a nationally representative randomized sample of 4,240 Canadian adults who are members of Angus Reid Forum, a national online sample of 50,000 Canadians used for political and other social polling”.**

e) a description of how participants were recruited,

**Response- The sampling frame is an existing Angus Reid Forum Panel (http://www.angusreidforum.com/), which is a national online sample of 50,000 adult Canadians used for political and other social polling (lines 60-61).**

f) descriptions of where participants were recruited and where the research took place

**Response- We have added this in lines 58-61, 87-90.**

Furthermore, please provide more information on the survey used. In the methods section, please refer specifically to the questions used to assess variables;

**Response- We have now added this in lines 95-106, 107-110.**

Discuss how items (for example, the choice of COVID-19 symptoms) were originated;

**Response- We have now added this in lines 83-84.**

and if you developed the questionnaire as part of this study and it is not under a copyright more restrictive than CC-BY, please include a copy, in both the original language and English, as Supporting Information.

**Response- We have now added the questionnaire as Supporting Information (S1 Appendix).**

Moreover, please include more details on how the questionnaire was pre-tested, and whether it was validated. "

**Response-The questionnaire was pre-tested as per standard polling procedures in about 60 people. Validation of symptoms was not possible at the time against antibody status but is planned in the main Ab-C study. We have added a line of pre-testing (lines 85-86).**

**Comment 6**- Please note that according to our submission guidelines (http://journals.plos.org/plosone/s/submission-guidelines), outmoded terms and potentially stigmatizing labels should be changed to more current, acceptable terminology. For example: “Caucasian” should be changed to “white” or “of [Western] European descent.

**Response 6- We have changed our terminologies from “Caucasian” to “white” and from “indigenous people” to “those of self-identified indigenous ethnicity”.**

**Comment 7-** Thank you for stating the following in the Financial Disclosure section: "PJ received funding from the Canadian Institutes of Health Research (https://cihrirsc.gc.ca/e/193.html; grant number: FDN 154277), and AR is funded by the Angus Reid Institute (http://angusreid.org/). The Angus Reid Institute collected the data used in this study and reviewed the manuscript." We note that one or more of the authors are employed by a commercial company: "deltaDNA Biosciences, Inc.,".

a) Please provide an amended Funding Statement declaring this commercial affiliation, as well as a statement regarding the Role of Funders in your study. If the funding organization did not play a role in the study design, data collection and analysis, decision to publish, or preparation of the manuscript and only provided financial support in the form of authors' salaries and/or research materials, please review your statements relating to the author contributions, and ensure you have specifically and accurately indicated the role(s) that these authors had in your study. You can update author roles in the Author Contributions section of the online submission form.

Please also include the following statement within your amended Funding Statement.

“The funder provided support in the form of salaries for authors [insert relevant initials], but did not have any additional role in the study design, data collection and analysis, decision to publish, or preparation of the manuscript. The specific roles of these authors are articulated in the ‘author contributions’ section.”

If your commercial affiliation did play a role in your study, please state and explain this role within your updated Funding Statement.

**Response 7a-“We have updated the funders. The commercial affiliation is only that for CB and no funding was involved. We have added the statement: “The funders provided support in the form of salaries for authors [DCW, HG], but did not have any additional role in the study design, data collection and analysis, decision to publish, or preparation of the manuscript. The specific roles of these authors are articulated in the ‘author contributions’ section.”**

**Comment 7b-** Please also provide an updated Competing Interests Statement declaring this commercial affiliation along with any other relevant declarations relating to employment, consultancy, patents, products in development, or marketed products, etc.

Within your Competing Interests Statement, please confirm that this commercial affiliation does not alter your adherence to all PLOS ONE policies on sharing data and materials by including the following statement: "This does not alter our adherence to PLOS ONE policies on sharing data and materials.” (as detailed online in our guide for authors http://journals.plos.org/plosone/s/competing-interests) . If this adherence statement is not accurate and there are restrictions on sharing of data and/or materials, please state these. Please note that we cannot proceed with consideration of your article until this information has been declared.

Please include both an updated Funding Statement and Competing Interests Statement in your cover letter. We will change the online submission form on your behalf.

Please know it is PLOS ONE policy for corresponding authors to declare, on behalf of all authors, all potential competing interests for the purposes of transparency. PLOS defines a competing interest as anything that interferes with, or could reasonably be perceived as interfering with, the full and objective presentation, peer review, editorial decision-making, or publication of research or non-research articles submitted to one of the journals. Competing interests can be financial or non-financial, professional, or personal. Competing interests can arise in relationship to an organization or another person. Please follow this link to our website for more details on competing interests: <http://journals.plos.org/plosone/s/competing-interests>

**Response 7b- We declare no competing interests.**

**Comment 8-** One of the noted authors is a group or consortium "Action to Beat Coronavirus in Canada/Action pour Battre le Coronavirus (Ab-C) Study Group". In addition to naming the author group, please list the individual authors and affiliations within this group in the acknowledgments section of your manuscript. Please also indicate clearly a lead author for this group along with a contact email address.

**Response 8- We have added the list of individual authors and affiliations within the group, "Action to Beat Coronavirus in Canada/Action pour Battre le Coronavirus (Ab-C) Study Group" in the Acknowledgement section, along with the lead author and contact email address.**

**Additional Editor Comments**

**Comment 1-** Abstract to be structured as per PLOS ONE policy.

**Response 1- We have structured the abstract as per PLOS ONE policy.**

**Comment 2-**Introduction should be more explicit, authors may wish to use pubmed https://pubmed.ncbi.nlm.nih.gov/?term=Determinants+of+self-reported+symptoms+and+testing+for+COVID-19 and other literature search platform.

**Response 2- We have revised the Introduction to be more explicit.**

**Comment 3-** There is a need to describe the outcome variables and the independent variables including plausible covariates, this will assist reader to understand the variables of interests for determinant of self-reported symptoms and testing for COVID-19.

**Response 3- We have now provided a description of the outcome and independent variables (lines 94-110).**

**Comment 4-** A bit of explanation on how data was managed to de-identify and assign unique identifier.

**Response 4- We have now added details on how data was managed to de-identify and assign unique identifier (lines 88-90).**

**Comment 5-** The statement of ethics should be very clear and the waiver reference number should be provided.

**Response 5- We have now added the research ethics statement and the ethics approval number (line 118-119).**

**Comment 6-** Include the statement for authors contribution.

**Response 6- We have now added the statement for authors’ contribution in the title page.**

**Reviewers’ comments**

Reviewer #1: REVIEWER COMMENT

The authors have presented a self-reported symptom and testing sampling of COVID-19 in Canada. Though a survey polling of this nature could come with some anecdotal exaggerations and biases, the study shows that the polling represents to a greater extent the Canadian census demographics.

MINOR COMMENTS

- The authors reported lower self-reported symptoms amongst the older age group, which they justified by the fact of differential presentation with dizziness and confusion, unlike the major listed symptoms for COVID.

- In addition, the authors reported “living in Quebec” (See line 133) as a determinant of “being tested”. It should be noted that Quebec has been the hardest hit province and this presupposes that the testing rates ought to be higher. If the authors want to make the assertion that “living in Quebec” is a determinant of “being tested”, they should try and run a regression with the testing rates and the number of reported COVID positive cases across the provinces when this data was collected. Otherwise, the authors may decide not to include that as a determinant.

**Response- We mention that testing rates are higher in Quebec in the discussion but do not believe it is appropriate to run a regression of testing data (which unlike this survey) are not representative versus symptom data.**

Overall, the presentation and the flow are generally good for the readers to understand.

Reviewer #2: Title:

1.The author may consider revising the title to predictors of self-reported symptoms and testing for COVID 19 in Canada using a nationally representative survey. In view of the depth of the statistical analysis done.

**Response 1- We have revised the title to predictors of self-reported symptoms and testing for COVID-19 in Canada using a nationally representative survey.**

Abstract:

1.The authors should consider including a sentence or two on COVID 19 as the opening narration of the abstract. This will bring to context the essence of the study.

**Response 1- We have now included a sentence on estimating the prevalence of COVID at the beginning of the abstract (lines 24-26).**

Introduction:

1. The introduction will benefit from additional information on the subject matter (COVID 19) and not just the expression of what the study was all about.

**Response 1- We have now added more information on COVID in the Introduction (lines 41-42).**

Materials and Methods:

1.The authors should consider defining what constitutes the outcome variable (COVID 19) symptoms (present or absent) properly in the methodology as it was done in the abstract (COVID symptoms, defined as fever plus difficulty breathing/shortness of breath, dry cough so severe that it disrupts sleep, and/or loss of sense of smell). The abstract is expected to represent an excerpt of the content of the manuscript.

**Response 1- We have now added the definition of COVID symptoms in the Methods section (lines 75-78).**

2.This manuscript will benefit from the details of how the survey weights was applied to the prevalence. Though a reference was provided but the cited document does not provide the needed clarity.

**Response 2- We have now added a statement of how weightings were applied to prevalence (lines 112-113).**

3.More information has to be provided by the authors on why IRB was not required for the study of this magnitude. It is also unclear what the statement "as per Unity Health Toronto Research Ethics Board" means. Was the ethics approval waived by unity Toronto Research Board? if so, reasons for such waiver have to be provided.

**Response 3- We have obtained IRB approval from Unity Health Toronto (lines 118-119)**

Results:

1. The information on the representativeness of the respondents by comparing the demographic characteristics with the Canadian population of 2019 will be more appropriate in the method as a standardization measure built into the study rather than having it in the results section.

**Response 1- We have now moved this to the Methods section.**

2.Table 1; it is unclear what gender constitutes others in the absence of an appropriate footnote.

**Response 2- We have now included a footnote to clarify what “other” gender constitutes.**

3. The authors should consider expunging the information on part of lines 133 to 115" Results using a narrower definition of COVID symptoms, namely having fever, difficulty breathing/shortness of breath, and severe dry cough were similar (data not shown) " as it is not adding any credence to the study.

**Response 3- We have now removed the statement.**

4.Table 2; the authors may consider revising the expression used for categorizing the outcome variable (COVID symptoms) from positive/negative to present or absent. This revision may bring clarity to the fact that these symptoms were not tested for but rather self reported.

**Response 4- We have now changed the expression from “positive/negative” to “present/absent”.**

Discussion:

1.The discussion will be benefit from some revision by bringing in other similar available studies though it is a common knowledge that there is a paucity of such studies. Efforts should be made to get some of those studies available so as to enrich the discussion.

**Response 1- We have made the point about such studies being required in the discussion and cited a recent NEJM paper.**
