# Supplementary material for: Predictors of self-reported symptoms and testing for COVID-19 in Canada using a nationally representative survey
Source: PLoS One. 2020 Oct 21;15(10):e0240778. doi: 10.1371/journal.pone.0240778 (PMC7577454; doi:10.1371/journal.pone.0240778)
Supplement: Attachment — Submitted filename: Review comments for PLoS One manuscript.docx [file pone.0240778.s004.docx]

The authors have painstakingly revised the manuscript in line with the review comments. However, the following revision will be required in order to make the manuscript publication worthy.

1. It is still unclear how the weighting of the sample size was done. This will require the authors to provide more details so as to bring clarity to it.

2. It is important for the authors to provide information on the source of the data collection instrument; was it adopted, adapted or developed for this study? Also, the method(s) of validating or assessing the reliability of the tool may be required. The statement on line 79 is unclear, which sampling frame were the authors referring to? The pretesting should not have been done among the same subjects who participated in the survey because it would have pre-empted them "The survey instrument was pre-tested in 60 individuals within the sampling frame."

3. It will also be good if the authors provide some sentence on the measures of used e.g. crude and adjusted odds ratio and the 95% confidence interval and rationale for using them in the data analysis section.

4. There is the need to provide footnotes for other ethnicity in tables 2 and 3.
