# Supplementary material for: Predictors of self-reported symptoms and testing for COVID-19 in Canada using a nationally representative survey
Source: PLoS One. 2020 Oct 21;15(10):e0240778. doi: 10.1371/journal.pone.0240778 (PMC7577454; doi:10.1371/journal.pone.0240778)
Supplement: Attachment — Submitted filename: Response to Reviewers.docx [file pone.0240778.s005.docx]

**Revision to submission [PONE-D-20-16713R1]: Predictors of self-reported symptoms and testing for COVID-19 in Canada using a nationally representative survey**

**Comment 1-** The authors have painstakingly revised the manuscript in line with the review comments. However, the following revision will be required in order to make the manuscript publication worthy.

It is still unclear how the weighting of the sample size was done. This will require the authors to provide more details so as to bring clarity to it.

**Response- We have now added a line stating that “The sample frame was established to match the Canadian census data from Statistics Canada.” (lines 58-59) under the Study design section. We have now also mentioned that “Since the sample frame matched the Canadian census data from Statistics Canada, no additional survey weight was applied.” (lines 109-110).**

**Comment 2-** It is important for the authors to provide information on the source of the data collection instrument; was it adopted, adapted or developed for this study? Also, the method(s) of validating or assessing the reliability of the tool may be required. The statement on line 79 is unclear, which sampling frame were the authors referring to? The pretesting should not have been done among the same subjects who participated in the survey because it would have pre-empted them "The survey instrument was pre-tested in 60 individuals within the sampling frame."

**Response- We have now added a line on the source of the data collection instrument stating that the questionnaire used for this study was developed based on expert opinion (line 80). We have clarified that the 60 people were tested prior to the main survey (our apologies for the earlier error) (line 83). The data collection instrument (questionnaire) was not “validated” as at the time of survey, there were no reliable seroepidemiological studies that related infection with symptoms (line 235-237). However, subsequent studies comparing antibody with symptoms which we defined as suggestive of COVID in our study confirmed that our choice of symptoms was appropriate. We have also mentioned this in the Limitations section (line 237-239).**

**Comment 3-** It will also be good if the authors provide some sentence on the measures of used e.g. crude and adjusted odds ratio and the 95% confidence interval and rationale for using them in the Analysis section.

**Response- We have now provided a sentence on the measure of outcome used (adjusted odds ratio and 95% confidence interval) and the rationale for using them in the Data analysis section (lines 106-108).**

**Comment 4-** There is the need to provide footnotes for other ethnicity in tables 2 and 3.

**Response- We have now included footnotes for “Others” ethnicity in tables 1, 2, and 3 listing the list of included ethnicities.**
